# Genetic variation in environmental enteropathy and stunting in Zambian children: A pilot genome wide association study using the H3Africa chip

**DOI:** 10.1371/journal.pone.0291311

**Published:** 2023-09-27

**Authors:** Monica N. Mweetwa, Talin Haritunians, Shishir Dube, Kanta Chandwe, Beatrice Amadi, Kanekwa Zyambo, Ta-Chiang Liu, Dermot McGovern, Paul Kelly

**Affiliations:** 1 Tropical Gastroenterology & Nutrition Group, University of Zambia School of Medicine, Lusaka, Zambia; 2 Department of Physiology, University of Zambia School of Medicine, Lusaka, Zambia; 3 F. Widjaja Foundation Inflammatory Bowel Disease Institute, Cedars-Sinai Medical Center, Los Angeles, California, United States of America; 4 Department of Paediatrics, University of Zambia School of Medicine, Lusaka, Zambia; 5 Washington University in St. Louis (WUSTL), St. Louis, Missouri, United States of America; 6 Blizard Institute, Queen Mary University of London, London, United Kingdom; Penn State: The Pennsylvania State University, UNITED STATES

## Abstract

**Purpose:**

Stunting is known to be heavily influenced by environmental factors, so the genetic contribution has received little attention. Here we report an exploration of genetic influences in stunted Zambian children with environmental enteropathy.

**Method:**

Children with stunting (LAZ < -2) were enrolled and given nutritional therapy. Those that were non-responsive to therapy were designated as cases, and children with good growth (LAZ > -1) from the same community as controls. Blood and stool samples were taken to measure biomarkers of intestinal inflammation, epithelial damage, and microbial translocation. Single nucleotide polymorphism array genotyping was carried out on saliva samples using the H3Africa consortium array.

**Results:**

Genome wide associations were analysed in 117 cases and 41 controls. While no significant associations with stunting were observed at P<5x10^-8^, likely due to the small sample size, interesting associations were observed at lower thresholds. SNPs associated with stunting were in genomic regions known to modulate neuronal differentiation and fatty acid biosynthesis. SNPs associated with increased microbial translocation were associated with non-integrin membrane ECM interactions, tight junctions, hemostasis, and G-alpha signalling events. SNPs associated with increased inflammation were associated with, ECM interactions, purine metabolism, axon guidance, and cell motility. SNPs negatively associated with inflammation overlapped genes involved in semaphoring interactions. We explored the existing coeliac disease risk HLA genotypes and found present: DQ2.5 (7.5%), DQ8 (3.5%) and DQ2.2 (3.8%); however, no children were positive for coeliac antibodies. We detected HLA-DRB:1301 and HLA-C:1802 with high odds ratios and P<0.05 in stunted children compared to controls.

**Conclusion:**

Genetic variations associated with stunting and the enteropathy underlying it, include variants associated with multiple pathways relating to gene expression, glycosylation, nerve signalling, and sensing of the nutritional and microbiological milieu.

## Introduction

Stunting is a form of childhood malnutrition which manifests as failure of linear growth [1] and is a major problem globally [2]. It is associated with increased mortality [3], impairment of neurocognitive development [4] and impaired responses to oral vaccines [5, 6]. For long it has been assumed, understandably, that malnutrition is the result of lack of nutritious food. However, there is now clear evidence showing that providing extra nutrients does not correct linear growth faltering in low- and middle-income countries [7]. Environmental enteropathy (EE), a diffuse inflammatory disorder of the small intestine, is likely to be one of the major obstacles [8–10]. Studies on stunted children have shown gut inflammation [11], maternal health [11], and enteropathogens in non-diarrheal stool [12] to be critical predictors of stunting. Recently the gut microbiome of malnourished individuals has gained interest as it has also been implicated in malabsorption [13].

The genetic contribution to malnutrition has not received much attention [14]. A search for genetic association studies in PubMed and Scopus (keywords childhood malnutrition, childhood stunting, genetics, Africa, Asia, India) found no genetic association studies for stunting. This reflects the received view that environmental factors are the main drivers of malnutrition. Epigenetic analysis has shown that methylation of key genes required for growth is modulated by undernutrition [15]. Schulze et al compared oedematous and non-oedematous SAM in Malawi and Jamaica and found hypomethylation across the genome [16]. Sickle cell disease is a common genetic disorder now known to be associated with malnutrition [17]. Transcriptomic analysis in children with environmental enteropathy and severe acute malnutrition has identified increased gene expression of NADPH oxidases and CXC chemokines, alongside reduced expression of nutrient absorptive and xenobiotic metabolising genes [18–20]. Genome wide association studies (GWAS) in disorders related to obesity (another form of malnutrition) has shown significant associations with multiple loci enriched in the central nervous system (CNS) [21–23]. Understanding the genetic contribution to stunting and its underlying enteropathy can help identify vital pathways that can be targeted, in addition to nutritional therapies, to improve the prospects of overcoming malnutrition.

Genomics has progressed greatly since the first human genome was sequenced, demonstrating the diversity of populations across the globe [24] and has provided a better understanding of medically relevant genetic variations. Despite this, historically most genotyping arrays did not provide adequate representation of non-European genomic variation. With the recent effort to characterise African genetic variation in depth, the H3Africa initiative [25–27] has led to the development of much more refined tools for analysing genetic variation, adapted to the range of variation observed on the continent. GWAS have been able to identify genetic associations with macro- and micronutrient intake in well-nourished individuals [28] with genome-wide significance. In this pilot GWAS, we set out to investigate the genetic contribution to stunting and EE using the H3Africa genotyping array [25] in stunted children and controls.

## Methods

### BEECH study

Children aged 0–18 months of age with stunting (length-for-age z score/LAZ of <-2 SD) or wasting (weight-for-length z score/WLZ of <-2 SD) were identified in four defined residential areas of Lusaka where children are at high risk of malnutrition and environmental enteropathy (Misisi, Chawama, Kuku and John Laing). They were invited to take part in the BEECH study, as previously described [29]. Briefly, 297 children were recruited between August 2016 and June 2019 and provided them with nutritional supplementation, including high energy protein supplement (corn-soy blend), a daily egg, and a multiple micronutrient sprinkle (Nutromix, Hexagon Nutrition, Chennai, India). These children were followed up to 24 months or for a minimum of 12 months. Children who failed to respond to nutritional rehabilitation were referred for medical evaluation and endoscopy was offered if no clinical explanation for stunting was found. Saliva samples were not collected from stunted children who did respond to nutritional rehabilitation. The study was approved by the University of Zambia Biomedical Research Ethics Committee (ref 006-02-16, dated 31^st^ May 2016).

### Recruitment and controls

Children with refractory stunting (cases) were defined as children from Misisi, Chawama, Kuku or John Laing in Lusaka with stunting (length-for-age *z* score (LAZ) -2 or less) which had not responded to the nutritional intervention over 4–6 months, and whose LAZ remained below -2. Controls were children from the same community whose LAZ was -1 or greater at recruitment. As this was a pilot study of samples obtained in an observational study, sample size calculations were not performed.

### Sample collection

Saliva was collected on the day of endoscopy using the Oragene DNA collection kit (DNAGenotek, Ottawa, Canada) in cases, and from controls at baseline. Blood and stool samples were collected at the same timepoints.

### Environmental enteropathy marker assays

Intestinal fatty acid binding protein (iFABP) was used as markers of epithelial cell damage. iFABP is a protein constitutively expressed in intestinal epithelial cells and released in circulation when gut epithelial cells are damaged. It was measured in plasma by ELISA (Cambridge Bioscience, Cambridge, UK).

Elevated levels of LPS, LPB and sCD14 in plasma indicated presence of microbial translocation resulting from impaired gut barrier function. Lipopolysaccharide (LPS) is a major molecule of the outer membrane of gram-negative bacteria which binds to lipopolysaccharide binding protein (LBP) and soluble CD14 (sCD14) in plasma. For LPS analysis, the Pyrochrome Limulus Amebocyte Lysate assay (Associates of Cape Cod, USA) kit was used. Human LBP and human sCD14 were assayed by ELISA (R&D systems, Minneapolis, USA).

Calprotectin and myeloperoxidase (MPO) were used as markers of inflammation in the gut. Faecal calprotectin signals the movement of neutrophils to the intestinal mucosa which release MPO as a defence mechanism. Faecal calprotectin was measured by ELISA (Immunodiagnostik AG, Bensheim, Germany) while faecal MPO was measured by ELISA (Epitope diagnostics, San Diego, CA, USA).

### Coeliac antigen assays

Anti-tissue transglutaminase (anti-TTG) was measured by ELISA (Inova Diagnostics, San Diego, CA, USA) for 158 plasma samples and anti-deamidated gliadin protein (anti-DGP) was measured by ELISA (Orgentec Diagnostika GmbH, Germany) for 72 samples.

### Genotyping, imputation, and Quality control (QC)

DNA was extracted from 164 saliva samples using the NucleoSpin Tissue kit (Macherey-Nagel) and genotyped using the H3Africa Consortium Array (Illumina) [25] following the manufacturer’s protocol at Cedars-Sinai Medical Center. Four samples failed genotyping and genotyping concordance for sample replicates was >99.98%. CookHLA software [30] was used to impute classical HLA alleles and SNPs within the HLA region of chromosome 6 following the default parameters and using a phased 1000 Genomes reference panel of 661 African individuals. Cases and controls were imputed together. Stringent sample and single nucleotide polymorphism (SNP) QC metrics were applied. Replicates, highly related samples and samples with gender discrepancies or >3% missing data (n = 6) were excluded. 158 DNA samples (117 cases and 41 controls) passed quality control and were available for downstream analyses. SNPs with >3% missing data, minor allele frequency (MAF) < 3%, or failing Hardy-Weinberg equilibrium (p<1x10^-6^) were excluded. All genomic coordinates are in GRCh37.

### Genetic association analysis

Genetic association for genotyped SNPs and the case-control binary variable was performed using logistic regression including principal components (PC) for population sub-structure, sex and age as covariates (PLINKv1.9) [31]. SNPs were considered suggestive if *P* < 1 x 10^−3^ and significant if P < 5 x 10−8. All SNPs with *P* < 1 x 10^−3^ were carried forward for functional annotation.

The chi-squared statistic was used to determine differences in frequency of imputed HLA alleles between cases and control. A *P* < 0.05 was considered significantly different (PLINKv1.9).

Association for quantitative biomarker variables was performed using linear regression including PC as well as case-control status as covariates (PLINKv1.9).). Additional analysis including sex and age did not significantly change the regression results therefore is not reported. Regression analysis for each quantitative biomarker phenotype was performed independentlybBetween 1–100 million permutations were performed for variants associated at p<1x10^-4^ (S1 Fig). SNPs were considered suggestive if permuted *P* < 1 x 10^−3^ and significant if permuted P < 5 x 10−8. All SNPs with permuted *P* < 1 x 10^−3^ were carried forward for functional annotation. LD clumping was performed to identify SNPs in linkage disequilibrium (r2>0.6) (—clump-r2 0.6—clump-p1 0.001 in Plinkv1.9).

SNPs are represented by reference SNP cluster IDs (rsid) in the manuscript. Those without this ID are represented by their genomic coordinates.

### Functional annotation

For biomarker SNP annotation, all SNPs associated with LPS, LPB and sCD14 were combined for functional analysis of microbial translocation whereas SNPs associated with calprotectin and MPO were combined to assess genetic contributions towards inflammation. iFABP was the only marker used to assess epithelial damage.

Initially, the SNPnexus software [32] was used to assess functional consequences of SNPs and gene mapping for suggestive SNPs. Genes within 50kb of a genomic locus were identified. These genes were then used for canonical pathway enrichment analysis using GENE2FUNC [33].

GWAS catalogue [34] was used for cross trait analysis of suggestive SNPs. Traits that were associated with suggestive SNPs at genome-wide significance (P<1e-8) were identified. SIFT [35] was used to predict consequences of non-synonymous SNPs on protein function using sequence homology and the physical properties of amino acids.

## Results

### BEECH study participants, phenotype, and controls

158 samples were studied in this pilot study. Controls were younger than cases as data were collected only at baseline, whereas samples from cases were collected at endoscopy when it was clear that the child had not responded to the nutritional intervention. Anthropometry demonstrates the difference between cases and controls, based on selection criteria. iFABP was higher in cases, and faecal calprotectin was lower (Table 1).

**Table 1:** Demographic and nutritional characteristics of cases and controls.

	Stunted children (n = 117)	Controls (n = 41)	*P*
Sex (M: F)	58:59	22:19	0.72
Age (months) [median/IQR]	18.0 (15.0,21.0)	4.0 (3.0,5.0)	**0.0001**
LAZ [median/IQR]	-3.27 (-3.89, -2.75)	-0.84 (-1.15, -0.37)	n/a
WLZ [median/IQR]	-2.25 (-2.70, -1.77)	0.13 (-0.54,0.33)	n/a
WAZ [median/IQR]	-0.74 (-1.30, -0.22)	0.77 (0.21,1.23)	n/a
HIV status			
Unexposed and uninfected	76	35	0.03
Exposed but un-infected	37	5	
Infected	2	1	
Plasma LPS (EU/ml) [median/IQR]	186 (0,327)	152 (122,247)	0.98
Plasma LBP (ng/ml) [median/IQR]	8.12 (5.66,13.62)	11.26 (8.82,14.89)	0.06
Plasma sCD14 (mg/l) [median/IQR]	1.63 (1.37,2.03)	1.40 (1.23,2.15)	0.36
Plasma iFABP (ng/ml) [median/IQR]	1922 (909,3128)	671 (258,1142)	**0.0001**
faecal MPO (ng/ml) [median/IQR]	143 (76,240)	173 (123,307)	0.15
faecal Calprotectin (mg/g) [median/IQR]	206 (121,389)	593 (266,1126)	**0.0001**

n/a: not appropriate, because statistical differences in anthropometric measures were not analysed as cases and controls were defined on this basis. Other differences were analysed using Fisher’s exact test or the Kruskal-Wallis test. LPS, lipopolysaccharide; LBP, LPS-binding protein; sCD14, soluble CD14 antigen; iFABP, intestinal fatty acid binding protein; MPO, myeloperoxidase.

### Genome wide survey of SNPs in case-control analysis

Initially, we set out to investigate variants that were significantly different between stunted children and controls. As no SNPs were significant at P < 5x10^-8^, we report the suggestive SNPs (*P* < 1 x 10^−3^) (S2 Fig). We found 248 SNPs which differed between cases and controls at this threshold (S1 Table). Analysis of all the possible consequences of these SNPs showed 264 consequences with the majority in intronic regions (S2 Table). One SNP in the coding region of the TGM3 gene (chr20:2312895:A/G:1) was non-synonymous resulting in an amino acid change at position 527 from asparagine to lysine however, this change is tolerated as predicted by SIFT. TGM3 is a transglutaminase involved in peptide crossing-linking and highly expressed in salivary glands, oesophagus, and the skin (Unirpot: Q08188)). Four SNPs chr3:169461571:A/C:1, chr3:169461571:A/C:1, chr4:82086779:A/G:1, and chr7:29010391:G/A:1 mapped to transcription factor binding site (TFBS) FOXJ2 (Q9P0K8), CUTL1 (P39880), FOXO4 (P98177), Meis-1 (O00470) respectively. Although TFBS do not directly affect the structure of proteins encoded, they can alter expression levels and/or translation rates. Interestingly, polymorphisms in the CUTL1 transcription factor binding site (TFBS) have been linked to growth delay in European individuals [36].

Nine known regulatory variants were suggestively associated with stunting (S2 Table). These were in the 3downstream, 3utr and 5upstream regions of gene involved in Fatty acyl-CoA biosynthesis, metabolism of carbohydrates and circadian clock regulation.

Cross trait analysis of SNPs associated with stunting using GWAS catalogue identified 12 SNPs previously associated with weight, schizophrenia, waist-to-hip ratio, and intake of total sugars among other disease in European populations (S3 Table). It was interesting to note that no SNPs observed in this exploratory study were not associated with any diarrhea gwas studies. There were no overrepresented pathways for genes overlapped or nearest to SNPs linked to stunting in our cohort.

### Expression quantitative trait loci analysis (eQTL)

Next, we set out to determine whether any of the SNPs had associations with gene expression levels previously reported [37] (GEO number GSE162630). This showed 3 SNPs (rs116508951: A>G, rs10971439: G>A and rs220179: A>G) strongly associated with increased expression of TMEM184A (P = 1.80E-10; FDR = 1.61E-04; FPKM = 31.1), PRSS3 (P = 2.75E-09; FDR = 1.34E-03; FPKM = 9.4), and RIPK4 (P = 8.09E-08; FDR = 0.0109; FPKM = 8.2) genes in duodenal biopsies, respectively (S4 Table) however, only rs220179 was linked to stunting (p = 0.04329). The rs116508951 and rs10971439 rs220179 are intronic variants whereas is a regulatory variant of RIPK4. PRSS3 (serine protease, mesotrypsin) is an important enzyme in intestinal physiology, involved in metabolism of water-soluble vitamins [38, p. 1], defensin processing [39] and activation of antimicrobial response by REG3 [40] while RIPK4 has been implicated in migration of pancreatic cancer cells by inhibiting the RAF1/MEK/ERK signalling repressor PEPBP1 [41]. RIPK4 is also associated with inflammatory gene expression and inflammatory cell death though its pathophysiological role is yet to be fully characterised [42]. TMEM18 acts as a binding site for heparin and is suggested to be linked with vesicular transport in exocrine excretion which increased body weight and reduced appetite in mice [43].

### Genome wide survey of SNPs in relation to biomarkers of enteropathy

Severe enteropathy has been reported [29] in biopsy samples of children in this cohort. Therefore, we set out to identify any variants that are significantly linked to markers of enteropathy. No significant associations were observed at P-perm<5x10^-8^, however several SNPs were associated at a lower significance level (P-perm<10^−3^) and are reported. This did not change when sex and age were considered. Table 2 shows the top SNPs identified at P-perm<5x10^-7^. All SNPs with P-perm<10^-3^are shown in supplemental tables.

**Table 2:** A summary of the biomarker GWAS analysis.

EED Feature	Biomarker^1^	H3Africa SNP ID (p<1e-7)	rsid	Nearest Gene^2^
Microbial translocation	**LPS**	kgp17071448	rs74541829	NA
		rs591274	rs591274	NA
	**sCD14**	kgp5394673	rs75074223	GNG2
Inflammation	**Calprotectin**	h3a_37_12_8474182_G_C	rs4483670	LINC00937
Epithelial damage	**iFABP**	kgp8391184	rs58389200	NA

^1^ Biomarkers that did not have any SNPs with p<1e-7 were excluded from this table.

^2^ These include genes within 50kb of the SNP.

#### Microbial translocation

1,109 SNPs were associated (S5 Table) with any of the biomarkers of microbial translocation (circulating LPS (698 SNPs), LBP (230 SNPs) and sCD14 (182 SNPs)); of these 87 were associated with decreasing microbial translocation. There was no overlap of SNP between biomarkers used (S3 Fig). Of the variants associated with increased concentrations of biomarkers of microbial translocation, four were non-synonymous variants; rs61747656, rs74082143, rs7226137, and rs115042205 with ‘moderate’ impacts on, SYNE3, HOXB1, and TRANK1 respectively, and with a deleterious effect on DCDC1 (S6 Table). rs8134061, was associated with increasing LPS and is known to be in eQTL for IFNAR1 in subcutaneous adipose (p = 1.8E-06) as reported in GTEx [dbGAP: phs000424.vN.pN] therefore is likely to be a modifier of IFNAR1 expression. One non-synonymous variant, rs2232613 in the coding region of the LBP gene (which changes proline to leucine) was negatively associated with plasma concentration of its gene product, the microbial translocation marker LBP (S5 Table). Greater than 50% of variants associated with decreasing microbial translocation were intronic.

rs1363119 which was associated with increasing LBP levels has previously been associated with gastroesophageal reflux in European populations (S7 Table). rs1739654, rs1383808 and rs9495528 SNPs which were associated with increasing LPS levels have previously been associated with IgG glycosylation, obesity-related traits, and urate levels in lean individuals respectively (S7 Table).

**Fig 1:**
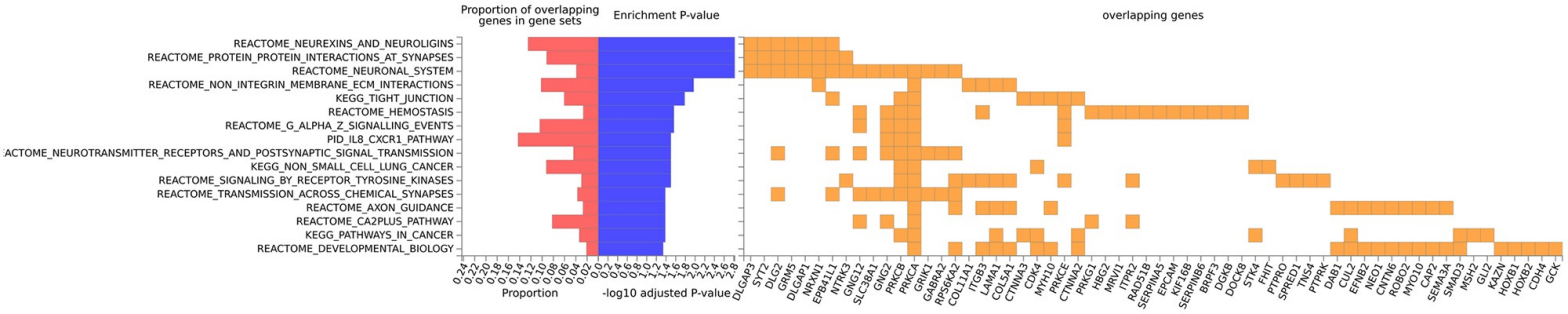
Pathways associated with increasing microbial translocation. The graph shows genes near SNPs linked to increased microbial translocation that significantly overlap functional pathways.

Functional analysis revealed genes within 50kb of variants associated with increased microbial to be involved in nuerexins and neuroligins interactions, protein-protein interactions at synapse, neuronal system, non-integrin membrane ECM interactions, tight junctions, hemostasis, G-alpha signalling events, CXCR1 synapse activity, among other pathways (Fig 1). LPS related genes accounted for most associated with nuerexins and neuroligins interactions, protein-protein interactions at synapse. LBP and sCD14 related genes did not show any significant pathways individually.

There was no significant pathway overrepresentation among genes related to decreased microbial translocation.

#### Intestinal Inflammation

1,153 SNPs were associated with biomarkers of inflammation (faecal myeloperoxidase and calprotectin) in this cohort (P <0.001) (S8 Table); of these 35 were associated with decreased inflammation. Nine SNPs (rs112410239, rs77204342, rs77563561, rs12525091, rs12338322, rs73096569, rs7424050, rs67243529, rs13007632) linked to MPO were also associated with increasing calprotectin (S3 Fig). Two of these SNPs close to LINGO2 and PTPRG genes were not in LD but reflect similar function in the cell cycle [44].

Of the SNPs associated with increased inflammation, two were synonymous mutations in the coding region of TESPAS1 (rs997173), and ORV51 (rs6930033) and 13 non-synonymous variants (S9 Table). All SNPs in coding regions associated with decreased inflammation were synonymous (S9 Table).

rs17041868 and rs16890640 have previously been associated with wait-to-hip ratio and urate level in overweight individuals respectively. GWAS catalogue also showed that 3 SNPs associated with increased calprotectin (rs17423748, rs56283067, rs12201899) have been linked to total body bone mineral density in individuals of mixed heritage (S7 Table). rs1846158, rs1325596 and rs1044299 SNPs associated with increased MPO have been associated with height in individuals of European ancestry (S7 Table).

**Fig 2:**
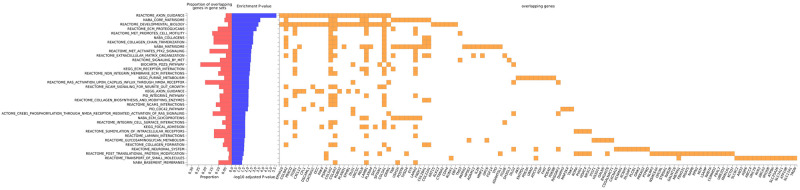
Canonical pathways associated with increased inflammation. The graph shows genes near SNPs linked to increased inflammation that significantly overlap functional pathways.

Positively associated variants mapped close to genes involved in axon guidance, core matrisome, developmental biology, ECM proteoglycans, MET cell motility, collagen chain trimerization, extracellular matrix organization and purine metabolism among other pathways (Fig 2). These pathways are largely driven by the genes close to variants linked to increased MPO (S9 Table).

Functional analysis of genes related to decreased calprotectin showed that SEMA6D and PLXNA4 were associated with other semaphorin interactions (S5 Fig).

#### Epithelial damage (iFABP)

756 SNPs were associated with circulating iFABP (S10 Table); of which 4 were associated negatively. Of the SNPs associated with increased epithelial damage, 7 non-synonymous SNPs in MTERFD3 (rs35548605), ACAD10 (rs35753710, rs75655687), CCDC135 (rs58373934), PIGN (rs9320001), MAML3 (rs115394118), and MEGF10 (rs17164935) are tolerated (S11 Table). There were no canonical pathways overrepresented in linked genes though 27 genes are involved in ion transport (S6 Fig). All variants negatively associated with epithelial damage were intronic and had no functionally significant pathways associated.

### Coeliac disease and HLA genes

As EE bears a moderate histological resemblance to coeliac disease, and we have previously reported [29] serological associations between histology and coeliac antibodies (though within the normal range), we imputed HLA genotypes to explore whether EE in these stunted children might be associated with the HLA haplotypes known to be associated with coeliac disease. The major risk haplotypes HLA-DQ2.5 (7.5% overall), HLA-DQ2.2 (3.8% overall) and HLA-DQ8 (3.5% overall) were all present in this cohort, but there was no significant difference between cases and controls. None of the children with HLA-DQ2.5, HLA-DQ2.2 or HLA-DQ8 were positive for anti-TTG or anti-DGP antibodies. Further, those children carrying coeliac disease genotypes did not have higher anti-TTG or anti-DGP concentrations (even within the normal range) in blood. Table 3 shows HLA genotypes with significantly different frequencies between cases compared to controls, notably HLA-DRB1:1301 HLA-DRB1:13, HLA-C:1802 and HLA-C:18 were higher in cases than controls.

**Table 3:** Summary of HLA alleles with significantly different frequencies in cases and controls.

CHR	SNP	Genotype	BP	F_Cases	F_controls	CHISQ	P	OR
6	HLA_DRB1_1301	HLA-DRB1	32660042	0.1033	0.0122	6.888	0.008675	9.332
6	HLA_DRB1_13	HLA-DRB1	32660042	0.1901	0.07317	6.213	0.01268	2.973
6	HLA_B_39	HLA-B	31431272	0.008264	0.04878	5.532	0.01868	0.1625
6	HLA_B_3910	HLA-B	31431272	0.008264	0.04878	5.532	0.01868	0.1625
6	HLA_B_35	HLA-B	31431272	0.03306	0.09756	5.428	0.01981	0.3162
6	HLA_B_3501	HLA-B	31431272	0.03306	0.09756	5.428	0.01981	0.3162
6	HLA_C_1802	HLA-C	31346171	0.08264	0.0122	5.015	0.02513	7.297
6	HLA_C_18	HLA-C	31346171	0.09917	0.02439	4.641	0.03122	4.404
6	HLA_B_07	HLA-B	31431272	0.05372	0.122	4.324	0.03758	0.4087

## Discussion

Stunting is a complex disorder which has several major contributors: poor nutrition, recurrent infection, and insufficient psychosocial stimulation [45]. Despite the numerous targeted interventions available, we are not able to effectively combat malnutrition globally, and thus mothers and children in low-income settings are particularly vulnerable [46]. Most research has centred around external risk factors of malnutrition while the host genome is rarely considered, and consequently there are few published studies with which we can compare our data [14]. In this pilot study, we attempted to identify polymorphisms significantly associated with stunting or EE in stunted children versus controls. This analysis was constrained by the small sample size of this pilot study, which was a subsidiary analysis of a larger study, and thus none of the associations reached genome-wide levels of significance (P<5x10^-8^). Moreover, identified associations do not necessarily equal causation, a significant limitation of genome-wide association studies [47]. Nevertheless, this study reveals the likely importance of several pathophysiological features of the response to malnutrition, and importantly suggests that genetic contributions to stunting may be of importance and deserving of further study. Some pathways of interest are highlighted below.

Glycosylation is an important post-translational (PTM) modification needed for proper folding and stabilization of the protein structure. In the gastrointestinal tract, this is a key feature of mucins that span the intestinal lining that allows them to be anchored in the cell membrane with better stability and form glycoprotein networks in the ECM which shield epithelial cells from microbes in luminal fluids. Decreased glycosylation is a common feature of immune-mediated GI disorders [48] and polymorphisms in glycosylation have been associated with ulcerative colitis [49]. Our findings show association of polymorphisms in this pathway with stunting and increased microbial translocation.

ECM communication between cells is also an important feature of barrier function that was associated with, increasing microbial translocation and inflammation. The ECM not only provides structural support for enterocytes but interacts with cells to guide functions such as stem cell self-renewal and differentiation. ECM-cell interactions are also implicated in inflammatory responses as integrins have been shown to recruit immune cells to affected sites [50]. A disturbed distribution of collagens and transglutaminases which are key in ECM formation is a feature of other GI disorders such as coeliac disease [51] and IBD [52].

Inflammation, as assessed by faecal biomarkers, was associated with SNPs close to genes implicated in purine metabolism, a key player in various inflammatory processes including modulation of regulatory T cells (Tregs) and Th17 cell functions [53]. Decreased inflammation was associated with semaphorin interactions and formyl peptide sensing. Extracellular guidance plays a key role in cell migration, an important part of immune response and semaphorins can act as repulsive or attractive signals for immune cell migration [54–56]. Microbial components are recognized by formyl peptide receptors which can also regulate stimulation of immune cells [56].

There is a plausible link between RIPK4 expression and rs220179 associated with stunting. RIPK4 is involved in stratified epithelial development and NF-κB activation which is key aspect of immune response towards foreign particles in the gut, especially microbes [57, 58]. We have previously witnessed high numbers of pathogens in this community of stunted children regardless of regardless if they experienced any diarrhoea in stools 7 days prior to sample collection [29]. Investigating polymorphisms correlated to RIPK4 expression as a proxy for immune regulation in the gut could elucidate genetic contributions toward EE adaptations to this high pathogen burden.

In the primary analysis–SNPs associated with stunting–the HLA region was not identified as harbouring polymorphisms of interest. However, in previous work we had identified high-normal coeliac disease antibodies as associated with enteropathy and hypothesized that coeliac risk genotypes could be present in this populations therefore decided to evaluate the HLA risk alleles. We detected HLA-DRB:1301 and HLA-C:1802 with high odds ratios and P<0.05 despite anti-TTG or anti-DGP concentrations being in the normal ranges. The lack of detection of coeliac disease in Zambian patients despite the presence of risk alleles is a question that needs to be explored further. This stunted cohort was relatively young with a median age of 18 months therefore, it is plausible that limited exposure to a diverse diet could mask the manifestation of coeliac disease.

The polymorphisms identified in this small genome wide survey do not yet yield information about gene function with sufficient clarity to permit an understanding of the pathophysiology of stunting, in the intestine or any other organ. For example, the identification of polymorphisms in CUTL1 TFBS related to stunting is intriguing, but it is not yet possible to say that the transcriptional programmes of the mucosa or the physiology of absorption is fixed according to CUTL1 mediated gene expression, or indeed in any other generalizable condition. Our data shows that there may be genetic influences on this very environmental condition, which probably means that host responsiveness to environmental stressors may be in part genetically determined. The data will hopefully also permit generation of hypotheses, enabling further work.

## Supporting information

S1 Fig QQ Plots of individual biomarker regression analysis. (TIF)

S2 Fig Manhattan plot of cases control regression analysis. The blue line represents the suggestive line adopted in this study (p<1e-3).
(TIF)

S3 Fig A venn diagram of total number SNPs associated with markers of microbial translocation at p<1e-3. (TIF)

S4 Fig A venn diagram showing total numbers of SNPs associated with markers of intestinal inflammation. Overlapping SNPs between biomarkers is shown in the intersections.
(TIF)

S5 Fig Pathway associated with decreased inflammation. (TIF)

S6 Fig Pathway overrepresented by gene set linked to increased epithelial damage. (TIF)

S1 Table Summary of regression analysis comparing SNP association with Cases against controls including genes within 50kb of SNPs. (CSV)

S2 Table Predicted functional consequences of SNPs associated with stunting. (CSV)

S3 Table Output of cross trait analysis of SNPs associated with stunting at p<1e-3. All SNPs shown had significance with traits at p<1e-8.
(CSV)

S4 Table Summary of eQTL analysis carried out on all genotyped SNPs. rs220179 was the only SNP also associated with stunting.
(CSV)

S5 Table Summary of regression analysis of SNP microbial translocation markers in plasma. This table includes genes nearest to SNPs.
(CSV)

S6 Table Consequences of SNPs associated with microbial translocation. (CSV)

S7 Table Summary cross trait analysis for SNPs associated with enteropathy at p<1e-3. All traits shown in this table had associations of p<1e-8.
(CSV)

S8 Table Summary of regression analysis of SNPs present and level of inflammation. This table includes genes nearest to SNPs.
(CSV)

S9 Table Consequences of SNPs associated with inflammation markers. (CSV)

S10 Table Summary of regression analysis of SNP and level of epithelial damage. This table also includes genes nearest to SNPs.
(CSV)

S11 Table Consequences of SNPs associated with epithelial damage. (CSV)
