# Supplementary figures and images for: Genetic variation in environmental enteropathy and stunting in Zambian children: A pilot genome wide association study using the H3Africa chip
Source: PLoS One. 2023 Sep 27;18(9):e0291311. doi: 10.1371/journal.pone.0291311 (PMC10529557; doi:10.1371/journal.pone.0291311)

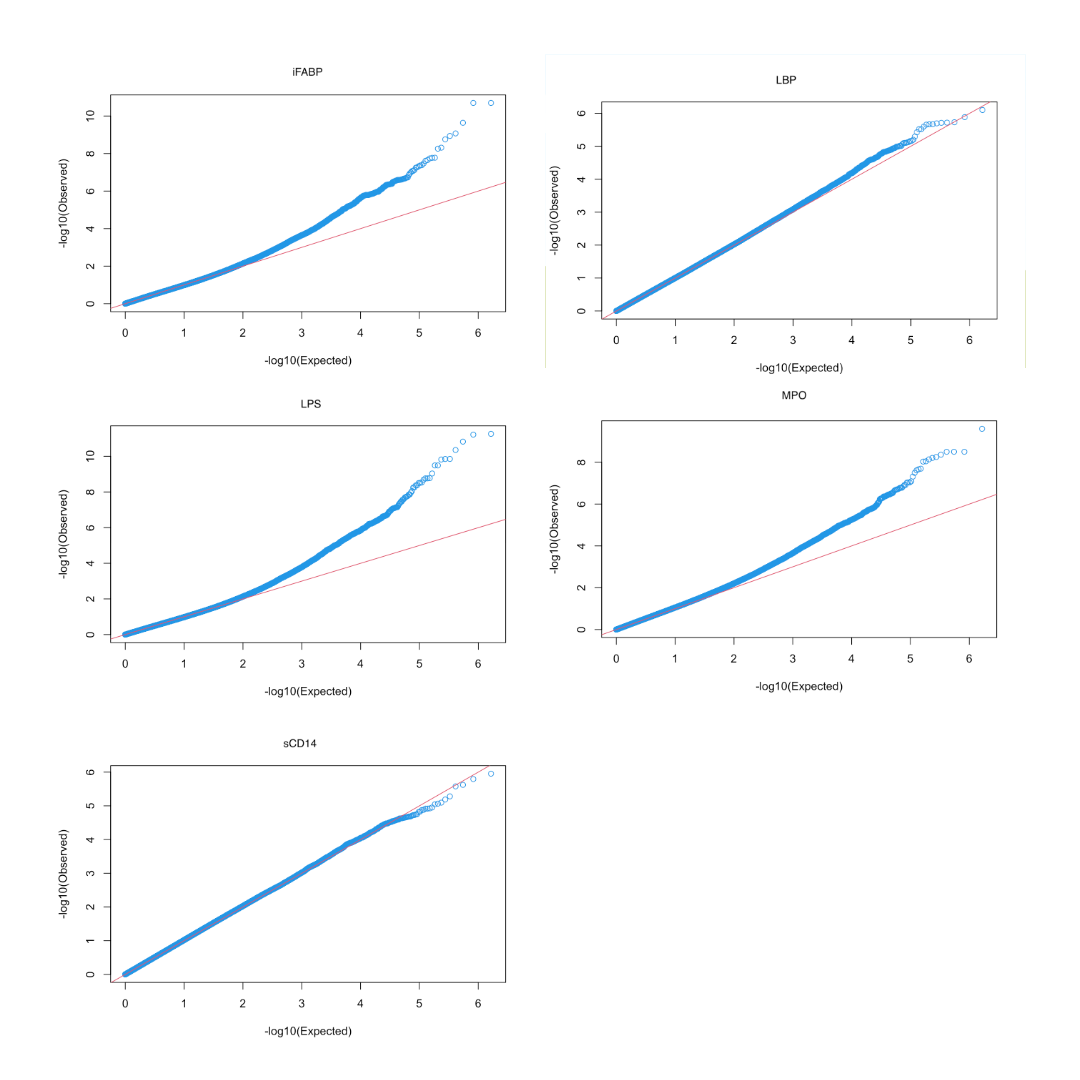

Supplement: S1 Fig — (TIF) [file pone.0291311.s001.tif]

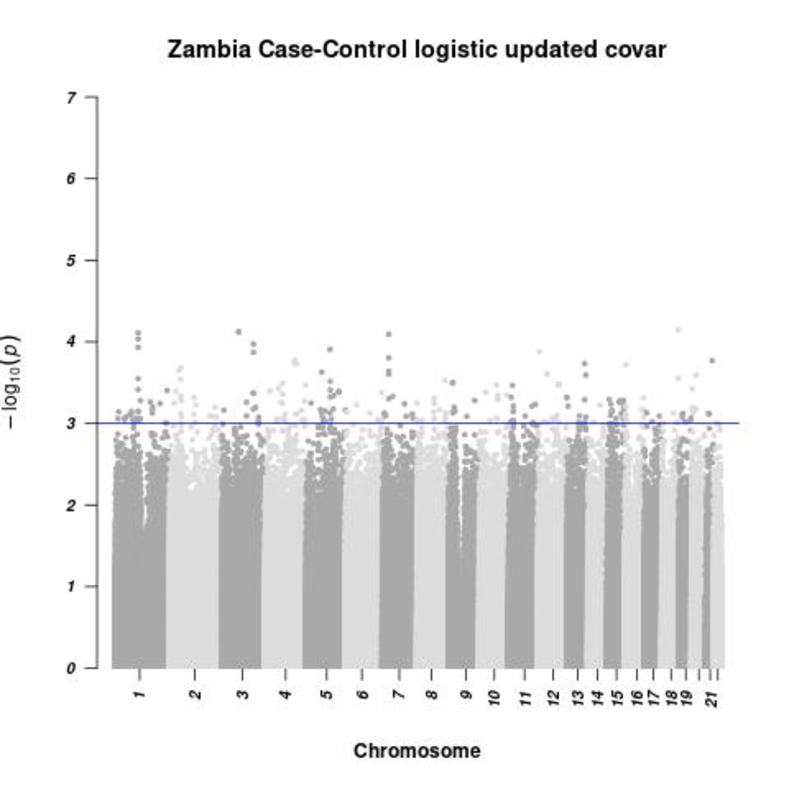

Supplement: S2 Fig — The blue line represents the suggestive line adopted in this study (p<1e-3). (TIF) [file pone.0291311.s002.tif]

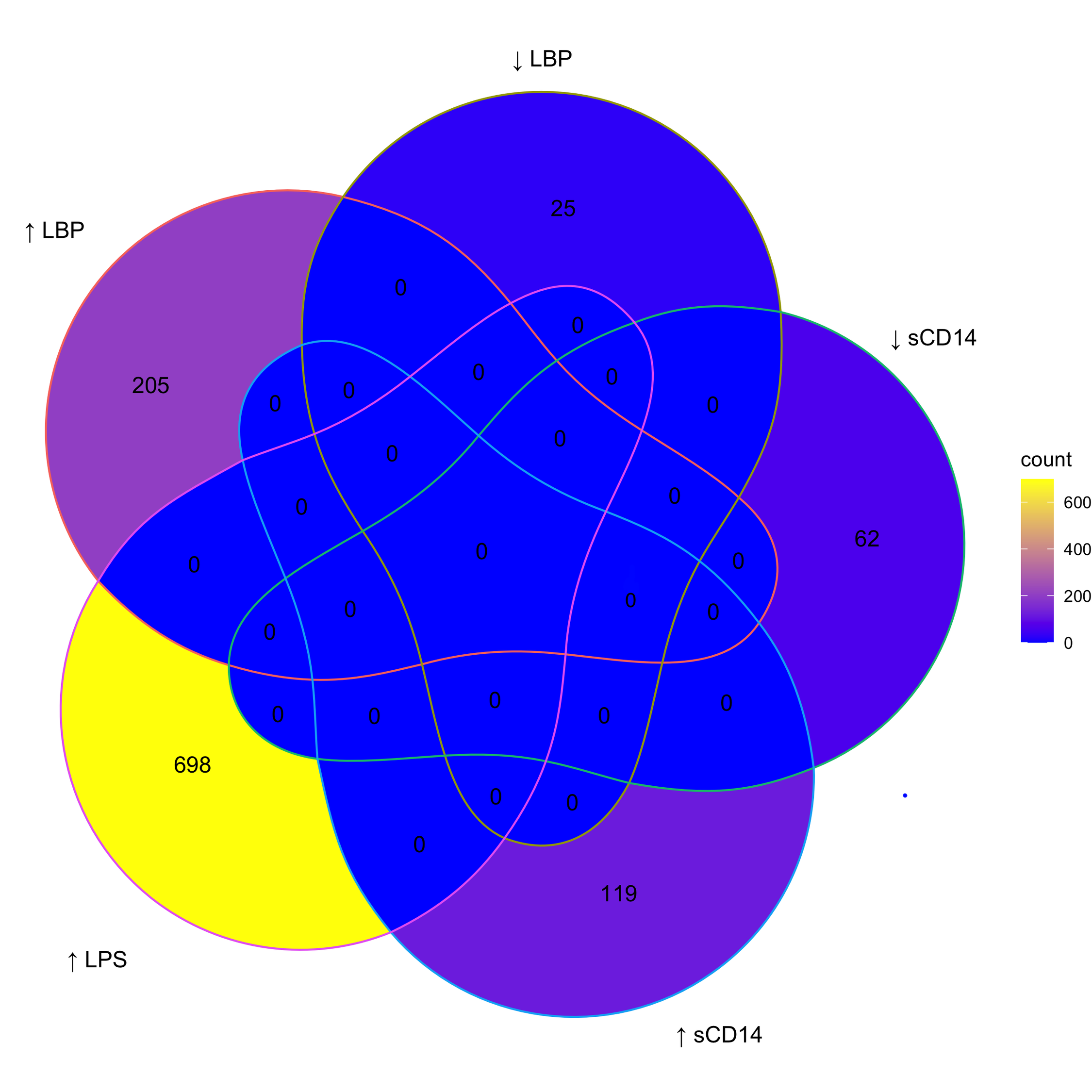

Supplement: S3 Fig — (TIF) [file pone.0291311.s003.tif]

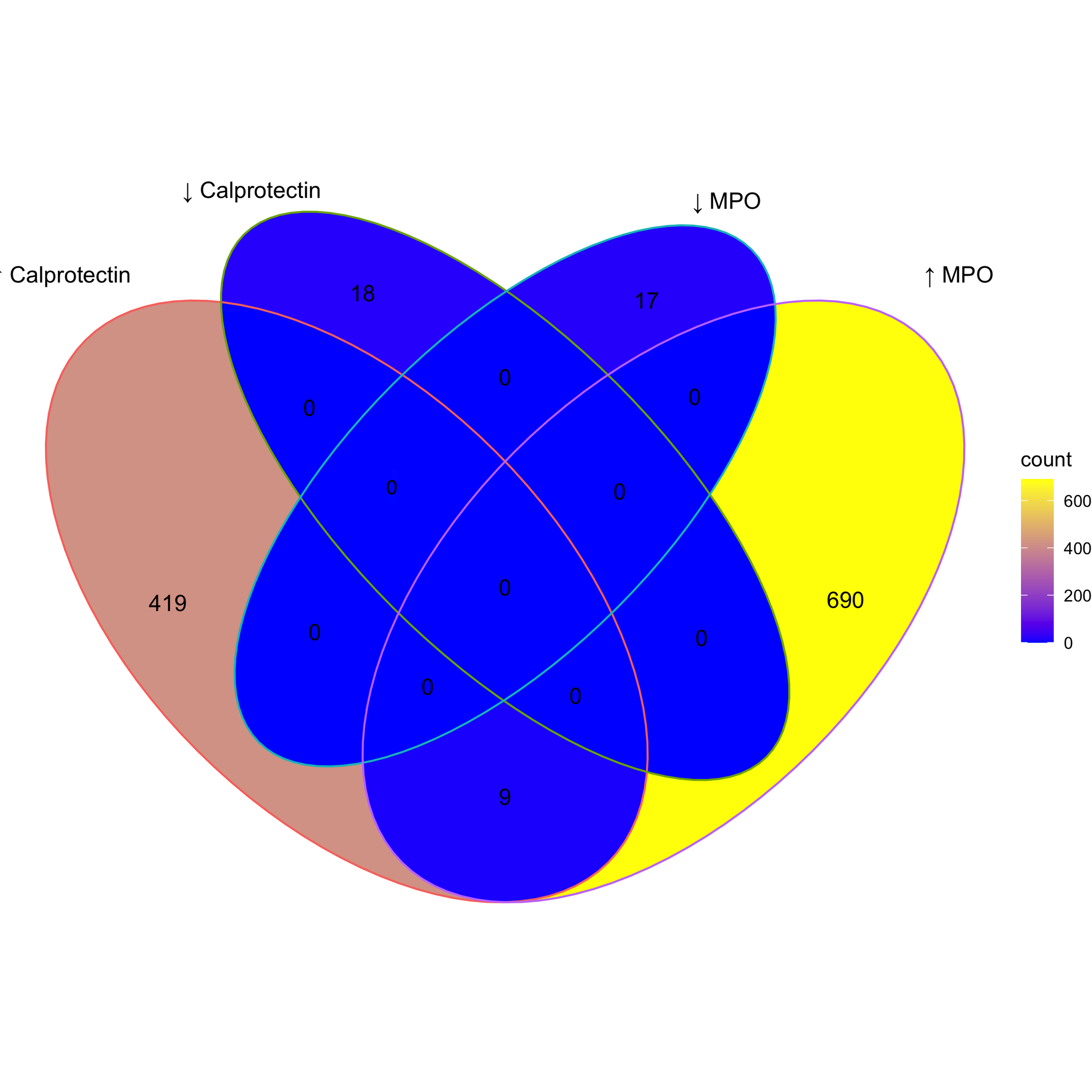

Supplement: S4 Fig — Overlapping SNPs between biomarkers is shown in the intersections. (TIF) [file pone.0291311.s004.tif]

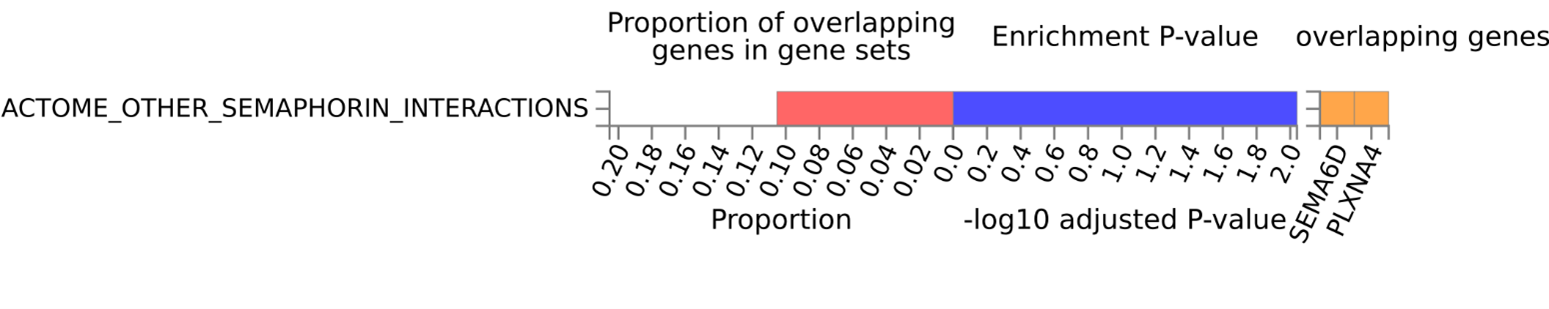

Supplement: S5 Fig — (TIF) [file pone.0291311.s005.tif]

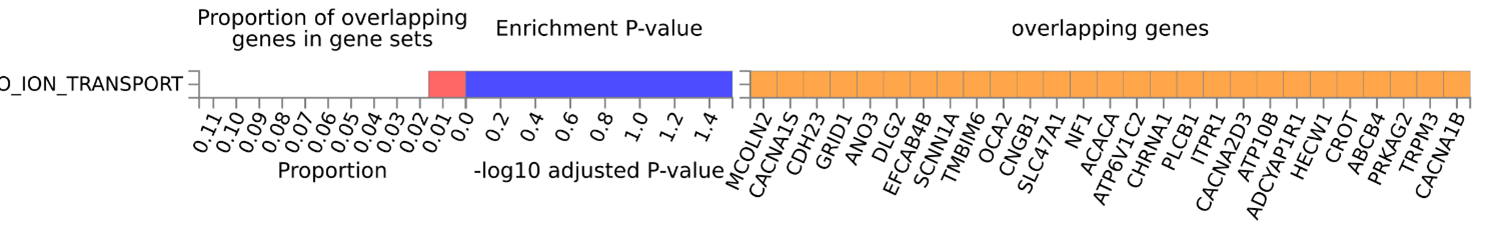

Supplement: S6 Fig — (TIF) [file pone.0291311.s006.tif]
